# Predictive value of persistent cerebrospinal fluid antibodies at 12 months for long-term clinical outcomes in pediatric anti-NMDAR encephalitis: a retrospective cohort study

**DOI:** 10.3389/fimmu.2026.1938331

**Published:** 2026-10-01

**Authors:** Hua Yang, Ruling Zhang, Jie Yang, Jingui Zhao, Xiaoling Zhong, Rong Luo

**Affiliations:** 1 Department of Pediatrics, West China Second University Hospital, Sichuan University, Chengdu, Sichuan, China; 2 Key Laboratory of Birth Defects and Related Diseases of Women and Children, (Sichuan University), Ministry of Education, Chengdu, Sichuan, China

**Keywords:** anti-N-methyl-D-aspartate receptor encephalitis, cerebrospinal fluid antibodies, children, clinical outcomes, prognosis

## Abstract

**Background and objective:**

Persistent cerebrospinal fluid (CSF) antibodies against the N-methyl-D-aspartate receptor (NMDAR) are common in children recovering from anti-NMDAR encephalitis, but their association with long-term clinical outcomes remains unclear. This study aimed to assess whether persistent CSF NMDAR antibodies at 12 months after diagnosis predict long-term clinical outcomes.

**Methods:**

In this retrospective observational cohort study, we included patients diagnosed with pediatric anti-NMDAR encephalitis between November 2012 and January 2026. Participants were stratified into two groups according to their CSF NMDAR antibody status at the 12-month follow-up. Clinical characteristics were compared between the groups, and long-term seizure-free rates were assessed using Kaplan–Meier survival analysis.

**Results:**

Among 162 initially identified patients, 41 (25.3%) met the inclusion criteria (mean age 8.7 ± 4.2 years; 78.0% female). At 12 months, CSF NMDAR antibodies remained detectable in 68.3% (28/41) of patients. Although the antibody-persistent group showed a trend toward a higher relapse rate than the antibody-negative group (42.9% vs. 0%), this difference did not reach statistical significance (*P* = 0.053). In contrast, the antibody-persistent group required significantly more frequent first-line (57.1% vs. 7.7%, *P* = 0.003) and second-line (39.3% vs. 0%, *P* = 0.024) immunotherapy.

Survival analysis revealed no statistically significant difference in seizure-free rates between the two groups during long-term follow-up (median 26 months; log-rank *P* = 0.601).

**Conclusion:**

In pediatric anti-NMDAR encephalitis, persistent CSF NMDAR antibodies at 12 months serve as a marker of incomplete remission and are associated with a trend toward a higher relapse rate and a significantly greater need for intensified immunotherapy, and may help identify patients who would benefit from more intensive immunomodulatory therapy.

## Introduction

Anti-N-methyl-D-aspartate receptor (NMDAR) encephalitis, which is characterized by psychiatric, motor, autonomic, and epileptic manifestations, is the most prevalent form of autoimmune encephalitis in children (1, 2). Diagnosis relies on detecting cerebrospinal fluid (CSF) IgG antibodies against the NMDAR GluN1 subunit, which directly impair synaptic function and promote neuronal hyperexcitability (3). Despite early immunotherapy leading to improved acute-phase outcomes, a substantial proportion of patients continue to suffer from long-term neurological sequelae, including disease relapse, incomplete functional recovery, and chronic epilepsy. Previous studies indicate that approximately 70-80% of patients with anti-NMDAR encephalitis develop acute seizures (4–6), with about 9-12% experiencing at least one disease relapse (7–9). Dynamic changes in CSF NMDAR antibody titers may reflect disease activity. Persistent CSF antibody positivity has been correlated with adverse clinical outcomes (10, 11). While Zhu H et al. (6) have indicated that early immunotherapy can improve seizure outcomes, it remains unclear whether persistent autoimmune activity contributes to sustained epileptic susceptibility. Additionally, the impact of CSF NMDAR antibody clearance on long-term epilepsy prognosis has not been adequately investigated in pediatric patients.

This retrospective cohort study evaluates the prognostic value of persistent CSF NMDAR antibodies at 12 months for long-term clinical outcomes in pediatric anti-NMDAR encephalitis, aiming to guide individualized clinical management.

## Methods

This retrospective cohort study was conducted at West China Second University Hospital, Sichuan University, and included pediatric patients diagnosed with anti-NMDAR encephalitis who were admitted for acute-phase management between November 2012 and January 2026. All patients underwent comprehensive diagnostic evaluation. Inclusion criteria were: (1) meeting Graus et al.’s diagnostic criteria (3); (2) complete clinical data; and (3) CSF NMDAR antibody testing at both diagnosis and 12-month follow-up. Patients lacking follow-up antibody testing or with incomplete data were excluded.

NMDAR antibodies were detected in CSF and serum using a cell-based immunofluorescence assay (Euroimmun AG, Lübeck, Germany), and results were interpreted qualitatively. Patients were categorized into two groups based on whether CSF NMDAR antibodies remained detectable at the 12-month follow-up: an antibody-persistent group (antibodies detectable at both diagnosis and the 12-month follow-up) and an antibody-negative group (antibodies no longer detectable at the 12-month follow-up). We retrospectively collected demographic, clinical, therapeutic, and relapse events. First-line immunotherapy included corticosteroids, intravenous immunoglobulins (IVIG), and plasma exchange (PLEX), administered either alone or in combination. Second-line immunotherapy, including rituximab, mycophenolate mofetil, and cyclophosphamide, was added in patients with an inadequate response to or dependence on first-line therapy.

Functional status was assessed using the modified Rankin Scale (mRS) (12); favorable outcome was defined as mRS ≤ 2, and unfavorable outcome as mRS > 2 (8). A delay of more than 4 weeks from symptom onset to the first immunotherapy was defined as delayed immunotherapy. Relapse was defined as recurrence of neurological symptoms after clinical improvement or stabilization lasting >2 months, or as symptom exacerbation (8). All reported epileptic events were confirmed by EEG, diagnosed only when accompanied by ictal or interictal epileptiform activity. Seizures occurring during follow-up were reviewed by neurologists and confirmed by correlating clinical semiology with the available EEG recordings. Given variability in CSF sampling times, results were categorized into standardized windows: 2-month (1–3 months), 6-month (4–8 months), 12-month (9–16 months), 24-month (18–28 months), and 36-month (30–42 months).

The study was approved by the Ethics Committee of West China Second University Hospital, Sichuan University (No. 2025-356). Informed consent was waived due to the retrospective design.

### Statistical analysis

Normality of continuous variables was evaluated with the Shapiro–Wilk test. Normally distributed data are reported as mean ± standard deviation (SD) and were compared using the independent t-test. Non-normally distributed data are expressed as median (interquartile range, IQR) and were compared using the Mann–Whitney U test. Categorical variables were analyzed using the chi-square test or Fisher’s exact test, as appropriate. Seizure-free survival was estimated with the Kaplan–Meier method, and between-group differences were assessed using the log-rank test. Statistical significance was defined as *P* < 0.05. All analyses were performed using SPSS (version 23.0) and GraphPad Prism (version 10.6).

## Results

### Demographic and clinical characteristics

A total of 162 patients were diagnosed with anti-NMDAR encephalitis between 2012 and 2026. After excluding 93 patients who lacked CSF NMDAR antibody testing at the 12-month follow-up and 28 patients with incomplete data, 41 patients (25.3%) were included in the final analysis. To evaluate representativeness, we compared baseline characteristics between included (n = 41) and excluded (n = 121) patients (**Supplementary Table S1**). No significant differences were found in age, sex, ICU admission, admission mRS, seizures, or relapse rate (all *P* > 0.05), indicating the included cohort was broadly representative. Patients were divided into two groups according to the CSF NMDAR antibody status at the 12-month follow-up: an antibody-persistent group (n = 28), in whom CSF antibodies remained detectable at 12 months, and an antibody-negative group (n = 13), in whom antibodies were no longer detectable. The mean age of the cohort was 8.7 ± 4.2 years, with a female predominance (78.0%) and a median follow-up duration of 26 months (IQR 22.5-47.0). Among patients with serial antibody testing, CSF NMDAR antibodies remained detectable in 95.0% (19/20) at 2 months, 92.0% (23/25) at 6 months, 68.3% (28/41) at 12 months, and 61.1% (11/18) at 24 months (**Supplementary Figure S1**).

No statistically significant differences were observed between the two groups in baseline demographics or acute clinical features, including the intensive care unit (ICU) admission rate (17.9% vs. 23.1%; *P* > 0.99), length of hospitalization (median 15 vs. 20 days; *P* = 0.207), CSF pleocytosis (25.0% vs. 30.8%; *P* = 0.993), abnormal brain MRI (53.6% vs. 53.8%; *P* = 0.987), and abnormal EEG findings (85.7% vs. 76.9%; *P* = 0.802). Serum NMDAR antibody status was available for all 41 patients at both diagnosis and 12-month follow-up. Positivity was similar between groups at diagnosis (64.3% vs. 53.8%; *P* = 0.524), but tended to be higher in the antibody-persistent group at 12 months (46.4% vs. 15.4%; *P* = 0.084) (**Table 1**).

**Table 1:** Comparison of clinical characteristics by CSF NMDAR antibody status at 12-month follow-up.

Characteristic	CSF NMDAR-Ab negative at 12mo (n=13)	CSF NMDAR-Ab positive at 12mo (n=28)	*P* value
Age, years (mean ± SD)	8.0 ± 4.1	9.4 ± 4.0	0.311
Female, n (%)	11 (84.6)	21 (75.0)	0.774
Median duration of hospitalization, days (median, IQR)	20.0 (13.0,25.5)	15.0 (9.0,20.8)	0.207
Teratoma, n (%)	0 (0)	1 (3.6)	>0.99
Prodromal symptoms, n (%)	6 (46.2)	8 (28.6)	0.453
Clinical features			
Psychiatric/Cognitive symptoms, n (%)	10 (76.9)	21 (75.0)	>0.99
Speech disturbance, n (%)	2 (15.4)	9 (32.1)	0.454
Sleep disorders, n (%)	6 (46.2)	10 (35.7)	0.524
Decreased level of consciousness, n (%)	2 (15.4)	8 (28.6)	0.600
Movement disorders, n (%)	5 (38.5)	15 (53.6)	0.368
Autonomic dysfunction, n (%)	5 (38.5)	10 (35.7)	>0.99
Auxiliary Examinations			
CSF pleocytosis, n (%)	4 (30.8)	7 (25.0)	0.993
Abnormal brain MRI at onset, n (%)	7 (53.8)	15 (53.6)	0.987
Abnormal EEG, n (%)	10 (76.9)	24 (85.7)	0.802
Serum NMDAR antibody positive at diagnosis, n (%) Serum NMDAR antibody positive at 12 months, n (%)	7 (53.8) 2 (15.4)	18 (64.3) 13 (46.4)	0.524 0.084
ICU admission, n (%)	3 (23.1)	5 (17.9)	>0.99
Mechanical ventilation, n (%)	2 (15.4)	4 (14.3)	>0.99
Relapse, n (%)	0 (0)	12 (42.9)	0.053
Admission mRS >2	11(84.6)	21(75)	0.774
12m mRS >2	1(7.7)	4(14.3)	0.930
Follow-up duration, months (median, IQR)	27 (24, 48)	24 (21, 46.75)	0.884

CSF, cerebrospinal fluid; SD, standard deviation; IQR, interquartile range; ICU, intensive care unit, mRS, modified Rankin Scale.

### Immunotherapy

First-line immunotherapy was administered to nearly all patients (92.7%), and the overall rate of first-line therapy did not differ between groups. Within the first-line regimen, the proportions of patients receiving corticosteroids, IVIG, IVIG plus corticosteroids, and PLEX were similar between the antibody-persistent and antibody-negative groups. In contrast, the antibody-persistent group significantly more often required repeated first-line courses (57.1% vs. 7.7%; *P* = 0.003) and second-line immunotherapy (39.3% vs. 0%; *P* = 0.024) (**Table 2**). Among second-line agents, rituximab was administered in 21.4% of antibody-persistent patients and in none of the antibody-negative patients, and mycophenolate mofetil in 28.6% versus 0%, with neither difference reaching statistical significance (*P* = 0.183 and *P* = 0.085, respectively); cyclophosphamide was rarely used (3.6% vs. 0%) (**Table 2**). These patterns indicate that persistent CSF antibodies were associated with escalation and intensification of immunotherapy rather than with a difference in initial first-line treatment. At the 12-month CSF sampling, ongoing maintenance immunosuppression was used in 4/28 (14.3%) patients in the antibody-persistent group and in none of the 13 antibody-negative patients (*P* = 0.288).

**Table 2:** Comparison of treatment strategies by CSF NMDAR antibody status at 12-month follow-up.

Characteristic	CSF NMDAR-Ab negative at 12mo (n=13)	CSF NMDAR-Ab positive at 12mo (n=28)	*P* value
First-line immunotherapy, n (%)	13 (100)	25 (89.3)	0.539
Steroids, n (%)	1 (7.7)	2 (7.1)	0.950
IVIG, n (%)	1 (7.7)	1 (3.6)	0.569
Steroids +IVIG, n (%)	11 (84.6)	22 (78.6)	0.975
PLEX, n (%)	4 (30.8)	11 (39.3)	0.858
Repeated first-line immunotherapy, n (%)	1 (7.7)	16 (57.1)	0.003
Second-line immunotherapy, n (%)	0 (0)	11 (39.3)	0.024
Rituximab, n (%)	0 (0)	6 (21.4)	0.183
Mycophenolate mofetil, n (%)	0 (0)	8 (28.6)	0.085
Cyclophosphamide, n (%)	0 (0)	1 (3.6)	>0.99
Time from onset to immunotherapy, days (median, IQR)	15.0 (6.5,21.0)	16 (11.25,34.5)	0.354
Delay immunotherapy, n (%)	5 (38.5)	14 (50.0)	0.491

CSF, cerebrospinal fluid; IVIG, IV immunoglobulins; PLEX, plasma exchange; IQR, interquartile range.

### Seizure outcomes

Seizure characteristics, including the occurrence of epileptic seizures, seizure as the initial symptom, seizure types, the incidence of status epilepticus, and the highest seizure frequency during the disease course, showed no statistically significant differences between the two groups (**Table 3**). Regarding anti-seizure medication (ASM) use, the most frequently prescribed agents in both groups were levetiracetam (35.7% vs. 23.1%) and valproate (21.4% vs. 38.5%), followed by clonazepam (28.6% vs. 23.1%) and oxcarbazepine (7.1% vs. 15.4%), with no significant between-group differences for any individual agent (*P* > 0.05 for all). The proportion of patients requiring ≥3 concurrent ASMs was numerically higher in the antibody-persistent group, although this did not reach statistical significance (17.9% vs. 7.7%; *P* = 0.702). Importantly, the proportion of patients continuing ASM treatment at the time of the last follow-up was comparable between the two groups (14.3% vs. 15.4%; *P* = 0.632) (**Table 3**). Kaplan–Meier analysis demonstrated no significant difference in long-term seizure-free survival between the two groups (log-rank *P* = 0.601; **Figure 1A**). To further explore potential determinants of long-term seizure outcome, we performed additional Kaplan–Meier analyses stratified by disease severity, patient age, and timing of immunotherapy. Seizure-free survival showed no significant association with disease severity (severe vs. non-severe; log-rank *P* = 0.581, **Figure 1B**), patient age group (young vs. adolescent; log-rank *P* = 0.859, **Figure 1C**), or delayed immunotherapy (log-rank *P* = 0.565, **Figure 1D**).

**Table 3:** Seizure outcomes according to CSF NMDAR antibody status at 12-month follow-up.

Characteristic	CSF NMDAR-Ab negative at 12mo (n=13)	CSF NMDAR-Ab positive at 12mo(n=28)	*P* value
Seizure as initial symptom, n (%)	6 (46.2)	12 (42.9)	0.843
Seizures, n (%)	12 (92.3)	23 (82.1)	0.702
Seizure type during disease course			
Generalized seizure, n (%)	9 (69.2)	15 (53.6)	0.344
Focal seizure, n (%)	4 (30.8)	8 (28.6)	>0.99
Status epilepticus, n (%)	7 (53.8)	14 (50.0)	0.819
Highest seizure frequency during disease course			
Single seizure, n (%)	1 (7.7)	3 (10.7)	>0.99
Multiple daily/daily, n (%)	8 (61.5)	13 (46.4)	0.368
Weekly, n (%)	1 (7.7)	4 (14.3)	0.930
Monthly, n (%)	1 (7.7)	3 (10.7)	>0.99
ASMs			
Levetiracetam, n (%)	3 (23.1)	10 (35.7)	0.493
Valproate, n (%)	5 (38.5)	6 (21.4)	0.280
Clonazepam, n (%)	3 (23.1)	8 (28.6)	>0.99
Oxcarbazepine, n (%)	2 (15.4)	2 (7.1)	0.579
ASMs ≥ 3, n (%)	1 (7.7)	5 (17.9)	0.702
ASM ongoing at last follow-up, n (%)	2 (15.4)	4 (14.3)	0.632

CSF, cerebrospinal fluid; ASMs anti-seizure medications.

**Figure 1:**
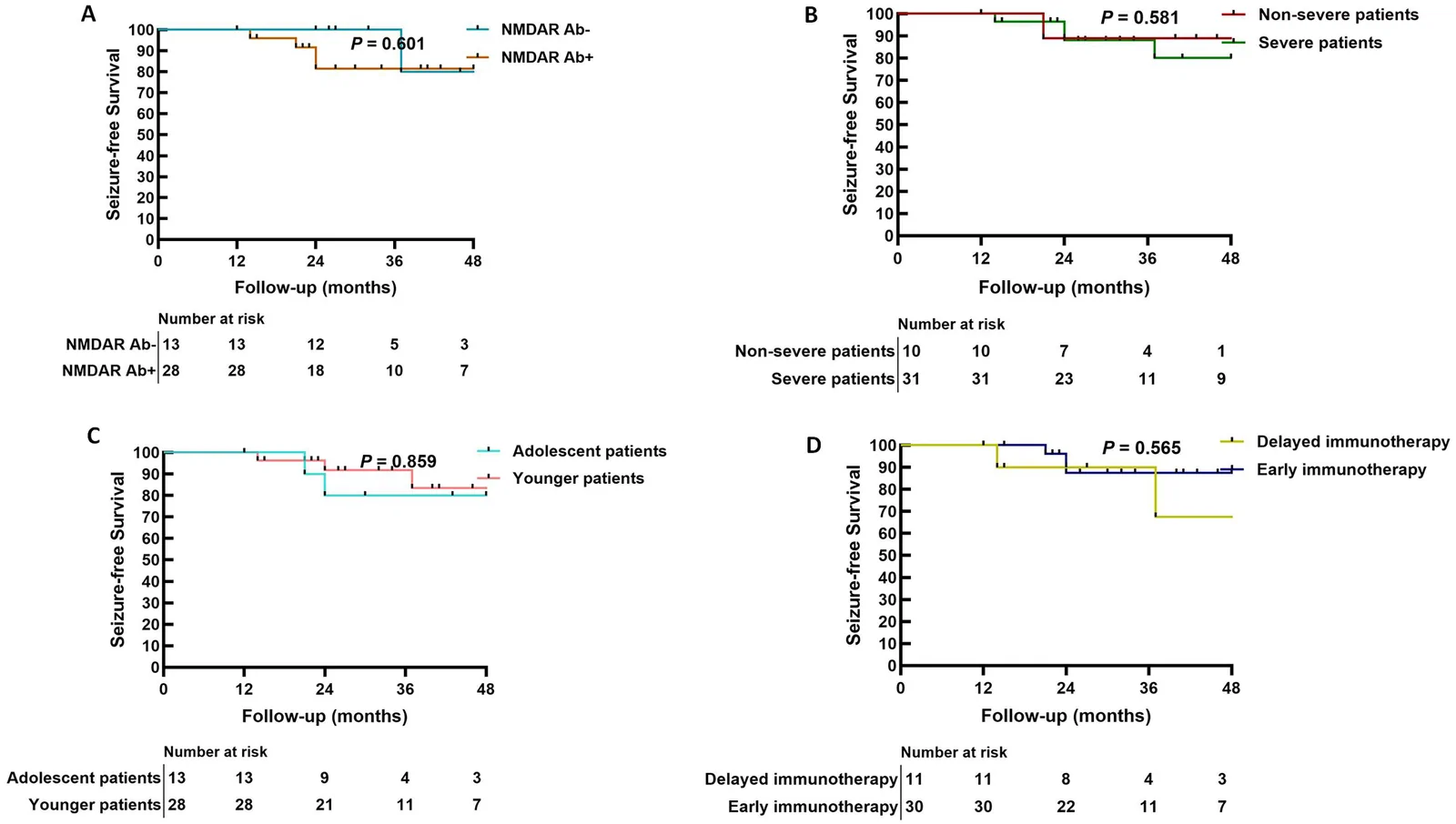
Kaplan-Meier analysis of seizure-free survival. **(A)** CSF NMDAR antibody-negative (dark blue) vs. positive (brown) at 12 months. **(B)** Non-severe (dark red) vs. severe (green) patients. **(C)** Adolescent (light blue) vs. younger (light red) patients. **(D)** Delayed (yellow) vs. early (purple) immunotherapy.

### Relapse

Relapse occurred in 12/28 (42.9%) patients in the antibody-persistent group and in none of the 13 antibody-negative patients (risk difference, +42.9%; *P* = 0.053). Follow-up duration did not differ significantly between the two groups (24 months [IQR 21.0–46.8] vs. 27 months [IQR 24.0–48.0]; *P* = 0.884) (**Table 1**).

## Discussion

Elevated antibody titres at diagnosis and during follow-up have been shown to correlate with disease severity and the likelihood of relapse in patients with anti-NMDAR encephalitis (13). However, the prognostic significance of sustained CSF antibodies during the recovery phase remains unclear. Our finding of persistent CSF NMDAR antibodies in 68.3% of children at 12 months aligns with the 60% rate reported by Ciano-Petersen et al. (14) in anti-NMDAR encephalitis patients. The two groups exhibited similar baseline characteristics, acute-phase clinical manifestations, and functional outcomes, which may reflect the generally favorable recovery potential in pediatric anti-NMDAR encephalitis. The plasticity of the developing nervous system could partially compensate for ongoing autoimmune injury, thereby supporting functional restoration. Moreover, the high rate of first-line immunotherapy administration (92.7%) in our cohort likely contributed to effective control of acute inflammation, establishing a foundation for neural repair.

This study revealed a trend toward a higher relapse rate in children with persistent CSF antibodies at 12 months (42.9% vs. 0%), although this difference did not reach statistical significance (*P* = 0.053), most likely attributable to limited statistical power given the small number of relapse events. Nevertheless, the antibody-persistent group required significantly more frequent first-line (57.1% vs. 7.7%, *P* = 0.003) and second-line (39.3% vs. 0%, P = 0.024) immunotherapy, indicating a greater burden of ongoing immunopathological activity and a heightened requirement for more targeted and potent therapeutic intervention. These observations are consistent with the notion of persistent CSF antibodies as a marker of incomplete remission and sustained B-cell or plasmablast activity (14). Although our data do not establish a statistically significant association between persistent antibodies and relapse, the marked increase in immunotherapy requirements supports the clinical relevance of CSF antibody persistence and suggests that longitudinal CSF antibody monitoring may help guide the intensity and duration of immunosuppressive therapy (8). Notably, the time from symptom onset to immunotherapy initiation and rates of treatment delay did not differ between groups, indicating that initial treatment timing is not a primary determinant of antibody clearance status at 12 months.

Repeated CSF sampling can be challenging to implement in routine pediatric practice, whereas serum sampling is more feasible. In our cohort, serum NMDAR antibody positivity was similar between groups at diagnosis (64.3% vs. 53.8%; *P* = 0.524) but tended to remain higher at 12 months in the antibody-persistent group (46.4% vs. 15.4%; *P* = 0.084), consistent with the CSF-based stratification. However, serum NMDAR antibodies are generally less specific than CSF antibodies and may not fully reflect intrathecal antibody synthesis (13), limiting their value as a surrogate for CSF monitoring. Serological monitoring may therefore complement, but not replace, CSF assessment.

Anti-NMDAR encephalitis is frequently associated with severe, often refractory seizures (15–17). However, our study found no significant differences in seizure characteristics between antibody-persistent and antibody-negative groups. Disease severity, patient age, and delayed immunotherapy also showed no significant association with seizure prognosis. Our results are consistent with the findings of Rada A et al. (18), who reported that while antibody titers are associated with acute seizure severity, they do not predict the development of chronic epilepsy, suggesting distinct pathophysiological mechanisms between the acute and chronic disease phases. Acute seizures are primarily driven by antibody-mediated NMDAR dysfunction and resultant neuronal hyperexcitability. After immunotherapy controls acute inflammation, residual antibodies may have limited epileptogenic potential. Chronic epilepsy appears more dependent on neural reorganization and synaptic plasticity following acute brain injury (19, 20). The implementation of a standardized anti-seizure medication regimen likely reduced the risk of seizures, while early immunotherapy may further suppress any residual immune-mediated activity (21, 22).

In our cohort, the rates of ASM continuation at the last follow-up were comparable between the antibody-persistent and antibody-negative groups (14.3% vs. 15.4%; *P* = 0.632), and no significant difference was observed in the use of any individual ASM. Although the antibody-persistent group showed a numerically higher rate of polytherapy with ≥3 ASMs (17.9% vs. 7.7%), this difference did not reach statistical significance. These findings align with the notion that standardized anti-seizure treatment, balancing adverse effects and efficacy, can effectively control seizures independent of the underlying autoimmune activity (23, 24). Indeed, seizures in anti-NMDAR encephalitis are generally responsive to conventional ASMs, and widely used first-line agents such as levetiracetam and valproate show no consistent superiority over one another in pediatric autoimmune encephalitis (25).

This study has several limitations. First, the single-center retrospective design inherently introduces selection bias. Second, the relatively small sample size, particularly in the antibody-negative group, may have affected the statistical power to detect significance for certain outcomes. Third, caution is warranted in interpreting the findings due to the variability in sampling timepoints. Fourth, NMDAR antibody results were analyzed qualitatively (positive/negative) without quantitative titers. Future studies with larger sample sizes and quantitative titer assessment are needed. Future prospective studies are needed to validate these results in larger cohorts.

In conclusion, this study provides new insights into the long-term management of pediatric anti-NMDAR encephalitis. The persistence of CSF NMDAR antibodies at 12 months was associated with a trend toward a higher relapse rate and a significantly greater need for first-line and second-line immunotherapy, underscoring the clinical relevance of CSF antibody persistence and its potential as a reference for guiding the intensity and duration of long-term immunomodulatory therapy. However, antibody persistence did not appear to be a major determinant of long-term seizure outcomes.

## Data Availability

The original contributions presented in the study are included in the article/**Supplementary Material**. Further inquiries can be directed to the corresponding authors.
