# Supplementary material for: Predictive value of persistent cerebrospinal fluid antibodies at 12 months for long-term clinical outcomes in pediatric anti-NMDAR encephalitis: a retrospective cohort study
Source: Front Immunol. 2026 Oct 1;17:1938331. doi: 10.3389/fimmu.2026.1938331 (PMC13668519; doi:10.3389/fimmu.2026.1938331)
Supplement: Supplementary file 1 [file SupplementaryFile1.docx]

Table S1 Comparison of baseline characteristics between included and excluded pediatric patients with anti-NMDAR encephalitis

| Characteristic | Included patients  （n=41） | Excluded patients  （n=121） | *P* value |
| --- | --- | --- | --- |
| Age, years (mean ± SD) | 8.9 ± 4.0 | 8.4 ± 4.0 | 0.468 |
| Female, n (%) | 32 (78.0) | 91 (75.2) | 0.713 |
| ICU admission, n (%) | 8 (19.5) | 40 (33.1) | 0.101 |
| Admission mRS >2, n (%) | 32 (78.0) | 102 (84.3) | 0.360 |
| Seizures, n (%)  Relapse, n (%) | 35 (85.3)  8 (19.5) | 94 (77.7)  13 (10.7) | 0.291  0.149 |

SD, standard deviation; ICU, intensive care unit
